# Inhalative Exposure to Vanadium Pentoxide Causes DNA Damage in Workers: Results of a Multiple End Point Study

**DOI:** 10.1289/ehp.11438

**Published:** 2008-07-31

**Authors:** Veronika A. Ehrlich, Armen K. Nersesyan, Christine Hoelzl, Franziska Ferk, Julia Bichler, Eva Valic, Andreas Schaffer, Rolf Schulte-Hermann, Michael Fenech, Karl-Heinz Wagner, Siegfried Knasmüller

**Affiliations:** 1 Institute of Cancer Research, Department of Medicine I, Medical University of Vienna, Vienna, Austria; 2 Austrian Workers Compensation Board, Vienna, Austria; 3 Department of Medicine II, Medical University of Vienna, Austria; 4 Commonwealth Scientific and Industrial Research Organisation, Human Nutrition, Adelaide, Australia; 5 Department of Nutritional Sciences, University of Vienna, Austria

**Keywords:** comet assay, cytokinesis-block micronucleus assay, DNA damage, occupational exposure, vanadium pentoxide

## Abstract

**Background:**

Inhalative exposure to vanadium pentoxide (V_2_O_5_) causes lung cancer in rodents.

**Objective:**

The aim of the study was to investigate the impact of V_2_O_5_ on DNA stability in workers from a V_2_O_5_ factory.

**Methods:**

We determined DNA strand breaks in leukocytes of 52 workers and controls using the alkaline comet assay. We also investigated different parameters of chromosomal instability in lymphocytes of 23 workers and 24 controls using the cytokinesis-block micronucleus (MN) cytome method.

**Results:**

Seven of eight biomarkers were increased in blood cells of the workers, and vanadium plasma concentrations in plasma were 7-fold higher than in the controls (0.31 μg/L). We observed no difference in DNA migration under standard conditions, but we found increased tail lengths due to formation of oxidized purines (7%) and pyrimidines (30%) with lesion-specific enzymes (formamidopyrimidine glycosylase and endonuclease III) in the workers. Bleomycin-induced DNA migration was higher in the exposed group (25%), whereas the repair of bleomycin-induced lesions was reduced. Workers had a 2.5-fold higher MN frequency, and nucleoplasmic bridges (NPBs) and nuclear buds (Nbuds) were increased 7-fold and 3-fold, respectively. Also, apoptosis and necrosis rates were higher, but only the latter parameter reached statistical significance.

**Conclusions:**

V_2_O_5_ causes oxidation of DNA bases, affects DNA repair, and induces formation of MNs, NPBs, and Nbuds in blood cells, suggesting that the workers are at increased risk for cancer and other diseases that are related to DNA instability.

Vanadium pentoxide (V_2_O_5_) is used for the production of metal alloys, for the manufacturing of lithium batteries and high-pressure lamps, and for the synthesis of chemicals [International Agency for Research on Cancer (IARC) 2006]. Its annual production worldwide is in the range of 60,000 tons (IARC 2006; Woolery 1997). Occupational exposure to the oxide occurs at production sites, during processing and refining of vanadium ores and sludges, during manufacturing of vanadium-containing products, in the course of combustion of vanadium-rich fuels, and by handling of catalysts in the chemical industry (Plunkett 1987). Environmental exposure to the metal and its oxides occurs via inhalation in the vicinity of metallurgical plants or through consumption of contaminated foods (Barceloux 1999; IARC 2006). Although foods contain low concentrations, nutrition is the major source of exposure for the general population (Barceloux 1999).

The National Toxicology Program (NTP 2002) found an increase of lung adenomas and carcinomas in mice after inhalative exposure to V_2_O_5_; this was paralleled by an increased incidence of hyperplasia in lung tissue. In male rats, the number of tumors was elevated (nonsignificantly), whereas in females no increase was found (IARC 2006; NTP 2002; Ress et al. 2003). These findings led to a reevaluation of the metal oxide (IARC 2006) and to its classification as a group 2B (“possible human”) carcinogen. One of the problems encountered in the risk assessment of V_2_O_5_ is the lack of human data. According to IARC (2006), inhalative exposure to V_2_O_5_ in vanadium plants exists worldwide, and several hundred workers may be affected; furthermore, workers of other industries may also be exposed. The occupational exposure limit in Austria for V_2_O_5_ in air is 0.05 mg/m^3^ (Bundesminister für Wirtschaft und Arbeit 2003). The Senate Commission of the German Research Foundation [Deutsche Forschungsgemeinschaft (DFG)] decided to suspend the maximum allowed concentration of V_2_O_5_ in workplace air because of its suspected carcinogenicity (DFG 2006). In the United States, the National Institute for Occupational Safety and Health and the American Conference of Governmental Industrial Hygienists established an occupational exposure limit of 0.05 mg/m^3^ air (Occupational Safety and Health Administration 2006). Measurements of air concentrations in vanadium factories yielded values in the range of 0.02–5 mg/m^3^ (IARC 2006).

Results of *in vitro* and animal studies indicate that the oxide causes formation of reactive oxygen species (Ingram et al. 2003, 2007; Wang et al. 2003; Zhang et al. 2001) and aneugenic effects (Migliore et al. 1993; Ramirez et al. 1997; Zhong et al. 1994) and interferes with DNA synthesis and repair (IARC 2006). Because DNA damage and aneugenic processes are known to play a role in the onset of human cancer (Duesberg et al. 2005; Pitot 1986), evidence of genetic damage in exposed humans would support the assumption of increased cancer risks. At present, only one study on the influence of occupational exposure to V_2_O_5_ on DNA stability has been published; in that study, Ivancsits et al. (2002) investigated DNA migration in leukocytes using the standard single-cell gel electrophoresis (SCGE) assay. The authors observed no indication of damage and found no elevation in the frequencies of sister chromatid exchanges (SCEs) or the concentration of 8-hydroxy-2′-deoxyguanosine in leukocytes. Lener et al. (1998) found no SCEs or chromosomal aberrations in blood cells of children living in the vicinity of a vanadium production site.

Our goal in the present study was to comprehensively evaluate the impact of inhalative V_2_O_5_ exposure on genetic stability. We monitored DNA migration in leukocytes of workers and matched controls with the standard SCGE assay, and we monitored oxidized bases using lesion-specific enzymes (Collins et al. 1993). Furthermore, we measured the sensitivity toward bleomycin (BLEO) and the repair of lesions induced by this cytostatic agent (Rajaee-Behbahani et al. 2001; Schmezer et al. 2001; Wei et al. 2005). BLEO sensitivity was initially monitored in chromosomal aberration assays (Hsu et al. 1989; Szekely et al. 2003) and later in SCGE experiments (Schmezer et al. 2001).

Additionally, we conducted cytokinesis-block micronucleus cytome (CBMN Cyt) assays with lymphocytes. This test is widely used for the detection of DNA damage in humans (Fenech 2007). Micronuclei (MNs), which are formed as a consequence of chromosome breakage and/or aneuploidy (Fenech and Morley 1985), correlate with the incidence of cancer in humans (Bonassi et al. 2007). Also, we evaluated the frequencies of nucleoplasmic bridges (NPBs) and nuclear buds (Nbuds) in lymphocytes. NPBs are assumed to occur when centromeres of dicentric chromosomes are pulled to the opposite poles of the cell at anaphase and provide a measure of chromosome rearrangements (Fenech 2006). Therefore, NPBs give direct evidence of genome damage resulting from misrepaired DNA breaks, which cannot be detected when MNs are scored as the only end point. Nbuds form as a consequence of gene amplification (Fenech 2006). Amplified DNA is selectively localized at specific sites of the nucleus and eliminated through recombinogenic events during the S-phase of mitosis (Shimizu et al. 1998, 2000).

Other parameters included in the present study were necrosis and apoptosis, and the nuclear division indices (Fenech 2006). We measured plasma concentrations of folate and vitamins B_6_ and B_12_ in both groups, because deficiencies of these micronutrients may increase MN levels (Fenech et al. 1997). Furthermore, we determined the plasma vanadium levels of the participants.

## Materials and Methods

### Study groups

We used the SCGE assay to study DNA migration in whole-blood leukocytes from 52 vanadium production workers exposed to V_2_O_5_ by inhalation and from 52 nonexposed controls (jail wardens). Additionally, we analyzed lymphocytes of 24 workers and 23 controls using CBMN Cyt experiments. We collected data concerning age, weight, height, and smoking status with a questionnaire (Table 1).

Workers are exposed to vanadium dust during the entire shift (8 hr) and are required to wear protective masks and gloves. The study was approved by the Ethics Committee of the Medical University of Vienna. After obtaining informed consent, we collected blood (2 ×10 mL) in heparin and EDTA tubes (Vacutainer-Systems, Becton Dickinson, Plymouth, UK). We stored blood samples at 4°C and transported them to the Institute of Cancer Research at the Medical University of Vienna. Whole blood was centrifuged at 623 ×*g* for 10 min at 14°C (Sigma Laboratory Centrifuge 4K15; Sigma Chemical Co., St. Louis, MO, USA) and plasma was collected, aliquoted, and stored at −80°C. We conducted experiments between October 2004 and May 2005.

### Exposure assessment

We determined vanadium concentrations in plasma using graphite furnace atomic absorption spectrometry, as described by Ivancsits et al. (2002), with Zeeman background correction at a temperature of 2,450°C. The calibration curves for vanadium were in the range of 0–40 μg/L, and the detection limit was 0.3 μg/μL (5.9 nmol/L).

### Measurements of vitamins B_6_ and B_12_ and folate

We determined pyridoxal phosphate (the active form of vitamin B_6_) in plasma by HPLC using commercial assays (Immundiagnostic AG, Bensheim, Germany) as described by Majchrzak et al. (2006). We measured vitamin B_12_ and folate in plasma using commercial radioimmunoassays (IUL Instruments GmbH, Königswinter, Germany) (Majchrzak et al. 2006).

### Leukocyte isolation and BLEO treatment for SCGE assays

We used the protocol of Buschini et al. (2002) for leukocyte isolation: EDTA-anticoagulated blood was maintained in erythrocyte-lysis buffer (155 mM NH_4_Cl, 5 mM KHCO_3_, 0.005 mM Na_2_EDTA, pH 7.4) at 37°C for 5 min; centrifuged (200 ×*g*, 5 min at 4°C); washed twice in phosphate-buffered saline (PBS; pH 7.4); and resuspended in RPMI 1640 (Sigma-Aldrich Chemie Gmbh, Munich, Germany) without serum (Buschini et al. 2002). We used a modified version of the protocol for BLEO sensitivity and DNA repair measurements (Schmezer et al. 2001). Leukocytes were treated with 10 μg/mL BLEO sulfate (Nippon Kayaku Co. Ltd., Tokyo, Japan) at 37°C for 30 min. To terminate the treatment, cells were washed twice in PBS (pH 7.4). To measure DNA repair capacity, we incubated a second batch of BLEO-treated cells for 15 min (37°C) before lysis and electrophoresis. Cell viability was monitored with trypan blue (Lindl and Bauer 1994). Viability of all samples was ≥ 90%.

### Alkaline SCGE (comet) assay

We performed the SCGE assay according to the guidelines of Tice et al. (2000). Briefly, cell pellets were mixed with 60 μL 0.5% low-melting-point agarose (Invitrogen Life Technologies Ltd., Paisley, Scotland), transferred to precoated (1.5% normal-melting-point agarose) glass slides, and sealed with a coverslip. The slides were placed on ice for 5 min to allow solidification of the agarose. After removing the coverslip, we immersed the slides in lysis solution (2.5 M NaCl, 100 mM Na_2_EDTA, 10 mM Tris, 1% Triton X, 10% dimethyl sulfoxide, pH 10.0) and kept them at 4°C for ≥ 1 hr. To prevent DNA damage, lysis and all subsequent steps were conducted under red light. After lysis, we placed the slides on a horizontal gel electrophoresis unit (C.B.S. Scientific, Solana Beach, CA, USA) and allowed the DNA to unwind at 4°C in alkaline electrophoresis buffer (300 mM NaOH, 1 mM Na_2_EDTA, pH ≥ 12.5) for 20 min. Electrophoresis was performed at 25 V and 300 mA for 20 min; then the slides were covered with neutralization buffer (0.4 M Trizma base, pH 7.5, 4°C) for 2 ×8 min and air dried. The coded slides were stained with ethidium bromide (20 μg/mL) and evaluated under a fluorescence microscope (Nikon 027012; Nikon, Tokyo, Japan). We used an automated analysis system (Helma and Uhl 2000) to acquire images, compute the integrated intensity profile for each cell, estimate the comet cell components, and evaluate derived parameters. For each experimental end point we analyzed three cultures, and we measured 50 randomly captured cells from each slide. To quantify DNA damage, tail lengths and tail moments were determined.

### Determination of oxidized purines and pyrimidines

We used a modified version of the protocol published by Collins et al. (1993) to measure endogenous formation of oxidized DNA bases. Formamidopyrimidine glycosylase (FPG) and endonuclease III (ENDO III) were provided by K. Angelis (Czech Academy of Sciences, Prague, Czech Republic).

After lysis of the cells, the slides were washed in enzyme buffer (0.1 M KCl, 40 mM HEPES, 0.5 mM Na_2_EDTA, 0.2 mg/mL bovine serum albumin, 4°C, pH 8) for 2 ×8 min. Subsequently, agarose-embedded cells were covered with 50 μL enzyme buffer or with enzyme solutions (1.0 μg/mL). Gels were sealed with a coverslip and incubated at 37°C in the dark for 45 min (ENDO III) or 30 min (FPG). Subsequently, the slides were transferred into alkaline buffer for unwinding, and electrophoresis was performed.

### CBMN Cyt experiments

We isolated lymphocytes according to the protocol of Fenech (2000). Briefly, Histopaque 1077 (Sigma) was overlaid with RPMI 1640–diluted blood and centrifuged at 318 ×*g* at 14°C for 30 min. Subsequently, the cell layer was removed, resuspended in RPMI, washed twice, and centrifuged (318 ×*g*, 14°C, 10 min).

We performed the CBMN Cyt test using the cytochalasin B technique described by Fenech (2007). We determined MNs, Nbuds, and NPBs, as well as apoptotic and necrotic cells, in samples from 24 workers and 23 controls using the cytome approach (Fenech 2007). Slides were evaluated by one of the authors (V.A.E.) who was trained in the laboratory of M.F. at Commonwealth Scientific and Industrial Research Organisation (Human Nutrition, Adelaide, South Australia), which has developed the CBMN Cyt assay and was centrally involved in the standardization of scoring criteria via the International Collaborative Project on Micronucleus Frequency in Human Populations (Human Micronucleus Project 2008). For each participant, we prepared lymphocyte cultures in duplicate. Each culture contained 10^6^ cells in 750 μL RPMI 1640 supplemented with 100 U/mL penicillin, 100 μg/mL streptomycin, 10% fetal bovine serum (Sigma), 2.0 mmol/L L-glutamine, and 30 μg/mL phytohemagglutinin (Invitrogen, Carlsbad, CA, USA). Cultures were incubated in round-bottom tubes (Becton Dickinson) at 37°C in a humidified atmosphere containing 5% CO_2_. Forty-four hours after mitogen stimulation, we added cytochalasin B (final concentration, 4.0 μg/mL; Sigma-Aldrich, St. Louis, MO, USA) to block cell division. The total incubation time of the cultures was 72 hr. Subsequently, the cells were harvested and used to prepare slides, which were air dried, fixed, and stained with Diff Quick (Dade Behring, Deerfield, IL, USA). From each participant, we scored 2,000 binucleated cells using a light optical microscope (Microphot-FXA; Nikon) (Fenech et al. 2003).

### Statistical analysis

We performed statistical analyses using Statistica 5.0 software (StatSoft Inc., Tulsa, OK, USA). The results are presented as medians and 25th–75th percentiles. All *p*-values were two-tailed, and we considered differences to be significant at *p* ≤ 0.05. We used the Mann-Whitney *U*-test for comparisons between exposed subjects and controls. We used Spearman’s correlation coefficients to test univariate relationships between different variables.

## Results

### Demographics of the study populations

The characteristics of participants are summarized in Table 1. Age and body mass index did not differ between the groups. All individuals were males and nonvegetarians. The control group included 11 smokers and the exposed group included 12. The concentrations of folate were similar in both groups, whereas levels of vitamins B_6_ and B_12_ were higher (8.1% and 31%, respectively) in the exposed group, possibly due to intake of supplements. Vanadium levels were 7-fold higher in the plasma of the exposed group.

### DNA migration in leukocytes

Table 2 summarizes the results of the SCGE experiments. We found no difference of DNA migration between exposed individuals and control subjects when the assay was carried out under standard conditions (which reflect formation of single- and double-strand breaks), but we observed increased tail lengths and tail moments in all other end points in the workers. Formation of oxidized purines (detected with FPG) was elevated by 7%, and the formation of pyrimidines (by ENDO III treatment) was enhanced by 33%. Furthermore, the sensitivity of the cells toward BLEO-induced DNA damage was higher (25%) in the exposed individuals. When we monitored DNA migration after a repair phase (15 min incubation after BLEO treatment), we observed a decrease of tail lengths (50%) and tail moments (57%) in the controls, whereas in the workers we observed no reduction of tail lengths and a less pronounced decrease of tail moments (30%).

### Frequencies of end points measured with the CBMN Cyt assay

Data on the frequencies of MNs, NPBs, Nbuds, apoptosis, and necrosis rates, as well as the division indices [nuclear divison index (NDI) and nuclear divison cyto-toxicity index (NDCI)] are summarized in Table 3. The number of MNs was 2.5-fold higher in the workers, and the numbers of NPBs and Nbuds were substantially increased (7-fold and 3-fold, respectively) .

To find out whether V_2_O_5_ exposure and smoking cause synergistic effects, we compared the frequencies of MNs and Nbuds in exposed smokers and nonsmokers to those in the corresponding controls. In nonexposed individuals, the levels (means ± SD) of MNs, Nbuds, and NPBs were similar in smokers and nonsmokers respectively (MNs, 1.77 ± 1.62 and 1.50 ± 1.86; Nbuds, 0.33 ± 0.39 and 0.36 ± 0.32; NPBs, 0.46 ± 0.81 and 0.45 ± 0.61). In workers, the MN rates were lower in smokers (4.17 ± 2.95 and 8.08 ± 4.54), whereas the frequencies of NPBs and Nbuds were more or less identical (NPBs, 6.83 ± 3.04 and 7.33 ± 2.50; Nbuds, 3.58 ± 2.76 and 3.30 ± 1.41).

The frequencies of necrotic and apoptotic cells were elevated in the workers by 55% and 50%, respectively; for the latter parameter the difference was not significant. The last two rows of Table 3 show the nuclear division indices. The division rates were similar in both groups, regardless of whether we considered the number of viable cells only (NDI) or also dead cells (NDCI). These results indicate that metal exposure has no significant impact on the proliferative capacity of viable or total lymphocytes, respectively.

## Discussion and Conclusions

In this article we present the results of the first comprehensive study on the impact of occupational exposure to airborne V_2_O_5_ on DNA stability. For seven of eight end points, we found significant differences between exposed workers and controls.

The most important observation is the 2.5-fold higher frequency of MNs in lymphocytes of the exposed individuals (Table 3), because a prospective study showed that MNs in peripheral blood lymphocytes are a valid biomarker for predicting an increased cancer risk in humans (Bonassi et al. 2007). Furthermore, a smaller study showed strong predictability of the MN biomarker in lymphocytes for cardiovascular disease risk (Murgia et al. 2007). Because the levels of folate and vitamins B_6_ and B_12_ were equal or higher in the workers (Table 1), we can exclude that the increased MN levels in this group are due to deficiencies of these micronutrients.

V_2_O_5_ and other vanadium compounds induce MNs in bone marrow cells of rodents (Leopardi et al. 2005; Sun 1996). Furthermore, DNA-damaging properties of vanadium compounds have also been found in human and animal cells *in vitro* (Ivancsits et al. 2002; Kleinsasser et al. 2003; Migliore et al. 1993; Ramirez et al. 1997; Rojas et al. 1996; Roldan and Altamirano 1990; Zhong et al. 1994).

In the present study, we observed an increase of other end points of chromosomal stability (NPBs and Nbuds) in the workers. The induction of NPBs and Nbuds was even more pronounced than the increase in MN levels (Table 3), and it is notable that a recent case–control study on smokers showed that elevated NPB and Nbud frequencies are more strongly associated with lung cancer risk than are MN rates (El-Zein et al. 2006).

We found no synergistic effect between smoking and V_2_O_5_ exposure in the present study. Bonassi et al. (2003) conducted a meta-analysis concerning the impact of smoking on MN frequencies among subjects in occupational and environmental surveys. They concluded that only nonexposed heavy smokers exhibit a significant increase of MNs, whereas the MN frequency was not influenced by smoking among individuals exposed to genotoxic agents. In the latter group, even slightly reduced MN levels were found in smokers compared with nonsmokers (Bonassi et al. 2003).

The only marker that was not elevated in the workers in the present study was DNA migration monitored in SCGE experiments under standard conditions, which reflect endogenous DNA damage such as single- and double-strand breaks and apurinic sites (Tice et al. 2000). This observation is in agreement with results of an earlier investigation (Ivancsits et al. 2002).

*In vitro* studies have shown that the metal oxide induces DNA migration in blood cells under standard conditions, but the doses required to cause effects were substantially higher than the plasma concentrations in the workers in the present study (Ivancsits et al. 2002; Kleinsasser et al. 2003; Rojas et al. 1996). Furthermore, results obtained in SCGE experiments with rodents indicate that vanadium compounds cause DNA migration in inner organs (Altamirano-Lozano et al. 1996, 1999; Leopardi et al. 2005; Villani et al. 2007).

It is notable that the basal levels for tail lengths are quite low in the present study. Our values are in a range similar to those found in other laboratories (Grossi et al. 2008; Undeger and Basaran 2005; Yoshida et al. 2006; Zhang et al. 2008) and also within the historical range of our laboratory (Bichler et al. 2007; Hoelzl et al. 2008; Steinkellner et al. 2005). Because we monitored the cell viability before electrophoresis, we can exclude that cell damage accounts for the low values. Furthermore, the results obtained with tail moment in the present study are essentially identical to those from tail length measurements.

Endogenous formation of oxidized DNA bases (shown by treatment with the repair endonucleases FPG and ENDO III) is higher in the exposed group. Earlier investigations found that vanadium-treated cells generate hydrogen peroxide and superoxide radicals (Huang et al. 2000; Shi and Dalal 1992; Ye et al. 1999; Zhang et al. 2001). These vanadium-induced radicals were shown to cause damage to macromolecules and lipid peroxidation (Donaldson et al. 1985), and it is conceivable that they account for the oxidative damage that we observed in the present study.

It is noteworthy that a number of human studies found increased sensitivity toward DNA damage by BLEO in individuals who are at increased risk for different types of cancer (Schmezer et al. 2001). In the present study, we found a strong difference between workers and controls after BLEO treatment and a 15-min repair phase (Table 2). Although the tail lengths decreased by 50% in the controls, we saw no reduction in workers after the repair phase, and the tail moments were decreased to a higher extent in the controls. This indicates that the metal oxide interacts detrimentally with DNA repair processes. Ivancsits et al. (2002) found severe inhibition of BLEO-induced repair by V_2_O_5_ in SCGE experiments with lymphocytes *in vitro*, whereas the repair of ultraviolet C–induced lesions was less affected. These findings can be taken as an indication that V_2_O_5_ inhibits pathways that are required for the repair of BLEO-induced lesions (homologous recombination repair, nonhomologous end joining, and base excision repair) (Schlade-Bartusiak et al. 2002; Schmezer et al. 2001; Wei et al. 2005) and, to a lesser extent, nucleotide excision repair, which is required to repair ultraviolet C–induced lesions (Wei et al. 2005).

As shown in Table 3, we found that the frequencies of necrotic cells were higher in the workers. Also, programmed cell death (apoptosis) was increased, but this effect did not reach significance. Induction of apoptosis by vanadium compounds has also been observed in earlier *in vitro* studies (Au et al. 2006; Huang et al. 2000; Lampronti et al. 2005; Ray et al. 2006; Wang et al. 2003; Ye et al. 1999), and it has been postulated that activation of mitogen-activated protein kinases by reactive oxygen species and/or by an oxidant-independent pathway may play a role (Chien et al. 2006; Choi et al. 2003; Huang et al. 2000; Luo et al. 2003).

Statistical analyses of the different end points showed no correlations between formation of oxidized purines and pyrimidines and MN induction (*p* = 0.3992 and 0.4679, respectively), which indicates that different mechanisms are involved. Although oxidative damage of DNA bases caused by V_2_O_5_ is probably due to release of reactive oxygen species, the formation of MNs may be due to its aneugenic properties, which have been found in *in vitro* studies (Galli et al. 1991; Roldan and Altamirano 1990; Zhong et al. 1994) and are apparently caused by disturbances of microtubule assembly (Ramirez et al. 1997).

We also failed to find correlations between vanadium plasma levels and formation of oxidized purines (*p* = 0.2340), pyrimidines (*p* = 0.2895), and MN frequencies (*p* = 0.1571). Earlier studies with workers exposed to metals other than vanadium indicate that polymorphisms in repair genes have a strong impact on MN formation (Iarmarcovai et al. 2006; Mateuca et al. 2005, 2008), and it is conceivable that they also play a role in the case of the effects caused by V_2_O_5_.

Overall, our results show that inhalative exposure to V_2_O_5_ increases the levels of oxidized bases and of MN, NPB, and Nbud frequencies in blood cells and affects their DNA repair capacity. It is notable that vanadium levels similar to or even higher than those found in our study have been detected in workers of other vanadium industries and in welders (Altamirano-Lozano et al. 1999; Huang et al. 2000; Ivancsits et al. 2002; Shi and Dalal 1992; Villani et al. 2007). Because the aforementioned parameters are causally related to diseases including cancer, our findings strongly suggest that more protective measures and periodical monitoring of the workers are required. Furthermore, the current exposure levels should be reduced to avoid health risks due to vanadium-induced DNA instability.

## Figures and Tables

**Table 1:** Distribution of selected characteristics in exposed subjects and controls.

Variable	Controls (*n* = 52)	Exposed (*n* = 52)	*p*-Value
Age (years)	38.0 (32.00–44.50)	43.0 (38.50–49.50)	> 0.05
Body mass index	27.0 (24.00–28.50)	26.0 (25.00–27.50)	> 0.05
Cigarettes/day (no.)	5.0 (0.00–17.00)	7.0 (0.00–21.00)	> 0.05
Vanadium (μg/L)	0.3 (0.24–0.39)	2.2 (1.54–3.89)	<< 0.0001
Folate (μg/L)	4.7 (3.04–6.40)	3.4 (2.54–4.205)	> 0.05
Vitamin B_6_ (ng/mL)	18.2 (14.38–21.55)	19.8 (14.06–36.94)	0.016
Vitamin B_12_ (ng/L)	665.0 (428.5–866.5)	968.0 (662.5–1215.0)	0.013

All study subjects were male Caucasians and nonvegetarians. Values shown are median (25th–75th percentiles).

**Table 2:** DNA migration in leukocytes of workers and unexposed subjects using different end points of the comet assay.

Test condition	Measure	Controls (*n* = 52)	Exposed (*n* = 52)	Δ° (%)	*p*-Value
Standard conditions	TL	4.3 (4.07–4.64)	4.1 (3.89–4.44)	−5	> 0.05
	TM	2.4 (2.27–2.79)	2.3 (2.16–2.59)	−4	> 0.05
FPG	TL	4.5 (4.21–4.80)	4.8 (4.14–5.39)	+7	0.0236
	TM	2.5 (2.34–2.62)	2.7 (2.27–3.24)	+8	0.0007
ENDO III	TL	7.3 (6.71–8.17)	9.7 (7.84–12.08)	+33	0.0019
	TM	3.5 (3.24–3.89)	3.8 (3.18–6.03)	+9	0.0023
BLEO	TL	13.2 (11.93–16.58)	16.5 (13.55–23.56)	+25	<< 0.0001
	TM	8.7 (7.55–11.72)	12.2 (10.07–15.98)	+40	<< 0.0001
BLEO + DNA repair	TL	6.6 (6.02–7.20)	17.8 (13.62–19.65)	+172	<< 0.0001
	TM	3.7 (3.41–4.20)	8.3 (7.10–11.39)	+124	<< 0.0001

Abbreviations: Δ°, difference between exposed subjects and controls; TL, tail length (μm); TM, tail moment. Values shown are median (25th–75th percentiles) of 150 measurements per person.

**Table 3:** Frequencies of micronucleated lymphocytes and total numbers of MNs, NPBs, Nbuds, and apoptotic and necrotic cells per 2,000 binucleated cells in workers and unexposed subjects.

End point	Controls (*n* = 23)	Exposed (*n* = 24)	Δ° (%)	*p*-Value
Total no. of MNs	2.0 (1.00–4.00)	5.0 (2.50–9.00)	+150	0.0132
Micronucleated cells	2.0 (1.00–4.00)	5.0 (2.50–8.00)	+150	0.0132
NPBs	1.0 (0.00–2.00)	7.0 (5.00–9.00)	+600	<< 0.0001
Nbuds	1.0 (0.00–1.00)	3.0 (2.00–5.00)	+200	<< 0.0001
Necrotic cells (%)	13.5 (10.7–16.5)	20.9 (19.4–25.9)	+55	<< 0.0001
Apoptotic cells (%)	3.0 (1.1–10.7)	4.5 (3.6–5.2)	+50	> 0.05
NDI	1.9 (1.81–1.94)	1.9 (1.86–1.96)	0	> 0.05
NDCI	1.7 (1.64–1.76)	1.7 (1.61–1.70)	0	> 0.05

Δ°, difference between exposed subjects and controls. Values shown are median (25th–75th percentiles).
