# Erratum

**Published:** 2009-01

In the article by Ehrlich et al. [Environ Health Perspect 116:1689–1693 (2008)], Kambis Atefie was inadvertently omitted from the list of authors. The correct list of authors and affiliations is as follows:

Veronika A. Ehrlich,^1^ Armen K. Nersesyan,^1^ Kambis Atefie,^1^ Christine Hoelzl,^1^ Franziska Ferk,^1^ Julia Bichler,^1^ Eva Valic,^2^ Andreas Schaffer,^3^ Rolf Schulte-Hermann,^1^ Michael Fenech,^4^ Karl-Heinz Wagner,^5^ and Siegfried Knasmüller^1^

^1^Institute of Cancer Research, Department of Medicine I, Medical University of Vienna, Vienna, Austria; ^2^Austrian Workers Compensation Board, Vienna, Austria; ^3^Department of Medicine II, Medical University of Vienna, Vienna, Austria; ^4^Commonwealth Scientific and Industrial Research Organisation, Human Nutrition, Adelaide, Australia; ^5^Department of Nutritional Sciences, University of Vienna, Austria

The authors apologize for the error.

